# Do Centenarians With Higher Life Satisfaction Live Longer?

**DOI:** 10.1093/geroni/igaa057.726

**Published:** 2020-12-16

**Authors:** Oscar Ribeiro, Lia Araújo, Rosa Marina Afonso, Laetitia Teixeira

**Affiliations:** 1 Department of Education and Psychology, University of Aveiro, Aveiro, Portugal; 2 Instituto Politécnico de Viseu, Viseu, Portugal; 3 Universidade da Beira Interior, Covilhã, Portugal; 4 ICBAS-UP, Porto, Portugal

## Abstract

Subjective well-being (SWB) is defined as a person’s cognitive and affective evaluation of life and has been recognized as one of the main psychological factors associated with better health and longevity in different age groups. Several studies evidenced its influence on all-cause mortality, but such a relation has been scarcely explored in individuals aged 100 years and over. The aim of this study is to evaluate the role of SWB in the survival of a sample of centenarians. Two studies conducted in Portugal (PT100 Oporto and PT100 Beira Interior) followed individuals from the age of 100+ years, checking their survival every six months over the period of December 2013 until June 2019. The Satisfaction With Life Scale (SWLS; Diener, Emmons, Larsen, & Griffin, 1985) was used at baseline as a measure of subjective well-being. Given that this is a self-reported scale, only a subsample of centenarians with cognitive capacity were included in this study (n=82; 67 (80.7%) women; mean age at baseline 101 years (sd=1.3 years)). Results obtained through a univariate Cox proportional hazards model suggest that longer survival was associated with higher levels of life satisfaction, highlighting the importance of this psychological dimension for longevity even at very advanced ages.

